# Seasonal Differences in Physiological Responses to Walking in Urban Parks

**DOI:** 10.3390/ijerph191912154

**Published:** 2022-09-26

**Authors:** Chorong Song, Harumi Ikei, Yoshifumi Miyazaki

**Affiliations:** 1 Department of Forest Science, Kongju National University, 54 Daehak-ro, Yesan-eup, Yesan-gun 32439, Chungcheongnam-do, Korea; 2 Center for Environment, Health and Field Sciences, Chiba University, 6-2-1 Kashiwa-no-ha, Kashiwa 277-0882, Chiba, Japan

**Keywords:** forest therapy, heart rate, heart-rate variability, physiological relaxation, preventive medicine, urban green space

## Abstract

The aim of the current study was to assess seasonal differences in physiological responses to walking in urban parks. In total, 51 Japanese male university students participated in this research. During each season, the participants walked for 15 min in an urban park and a city area, which was used as the control site. Heart-rate variability and heart rate were used as physiological indicators. The mean values of each indicator in a comparison between walking in an urban park and a city area were compared according to each season. In addition, to show the physiological effect of walking in an urban park, differences (between walking in an urban park and walking in a city area) were calculated. Then, differences according to each season were compared. The results showed that the participants had increased parasympathetic nervous system activity in all seasons except summer. Moreover, they had decreased sympathetic nervous system activity in spring and fall and decreased heart rate in all seasons. Compared with walking in urban parks in summer, walking in urban parks in spring, fall, and winter had a greater relaxation effect on parasympathetic and sympathetic nervous system activities; hence, the physiological effects of walking in urban parks vary based on season.

## 1. Introduction

In modern times, people commonly spend their daily lives sitting indoors [1], and the number of populations with insufficient physical activity has increased [2]. This type of lifestyle has caused significant health issues. If this problem is not immediately resolved, people will be at high risk of metabolic diseases, and they can develop different chronic diseases, such as diabetes and cardiovascular conditions [3,4,5].

Since the importance of physical activity is emphasized [6], the role of urban green spaces in promoting the health and well-being of humans has received increasing attention. Urban green spaces are the most accessible natural environments for people in modern times. Moreover, they have important social, physical, and mental benefits that enrich the lives of urban residents [7,8,9,10,11].

Previous studies have been conducted to assess the advantages of the natural environment. Walking in urban forests has a restorative effect countering psychological stressors or mental fatigue [12,13,14] and can improve mood and cognitive function [15,16]. Epidemiological research has revealed a positive association between exposure to urban green spaces and the perceived general health and longevity of residents [17,18,19]. Moreover, physical activities, such as brief walking in an urban park, have physiological relaxation effects and psychological advantages. For example, they can improve mood states and relieve anxiety [20,21,22]. Recent studies have evaluated the increasing value of urban green spaces for human benefits from different perspectives, such as socioeconomic welfare [23,24] and ecosystem services [25]. However, research about the effect of direct use among humans, such as spending time in urban green spaces, may be insufficient.

Previous reports, which used similar experimental design and locations, partly examined the effects of walking in urban parks in spring [20], fall [21], and winter [22]. These studies showed that walking in urban parks has physiological and psychological benefits, and that the effects differed according to season. However, there is no study comparing differences in the effects of walking in urban parks according to season.

The thermal environment is expected to change along with seasonal changes, and it has a significant impact on the human body’s response. In winter, the body’s stress response caused by the cold can be reduced by wearing clothes, but in summer, the outdoor heat cannot be controlled. Thus, the relaxation effect of walking in the urban park is expected to be lower in summer than in other seasons. Therefore, the aim of this study is to assess seasonal differences in physiological responses to walking in urban parks. A new experiment was performed in the summer, and data analysis was conducted using the previously reported spring [20], fall [21], and winter [22] data.

## 2. Materials and Methods

### 2.1. Experimental Sites

**Figure 1:**
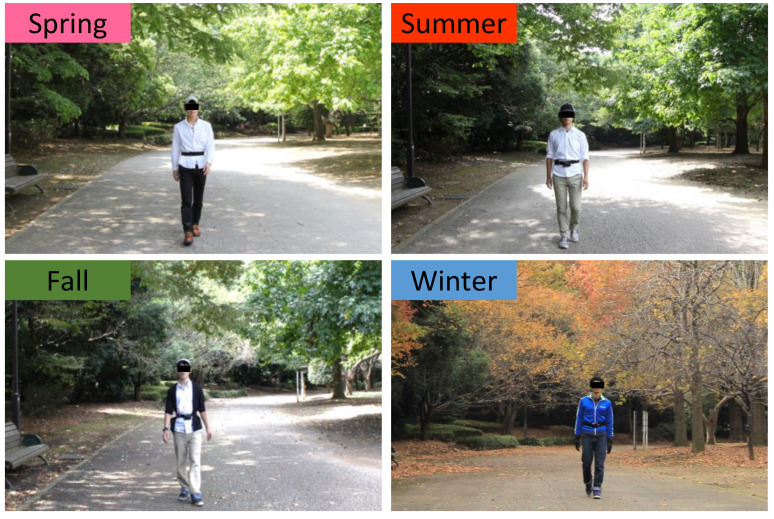
Experimental scene in the urban park. The top left photo (spring), bottom left photo (fall), and bottom right photo (winter) are reprinted from Song et al. (2014) [20], Song et al. (2015) [21], and Song et al. (2013) [22], respectively.

**Figure 2:**
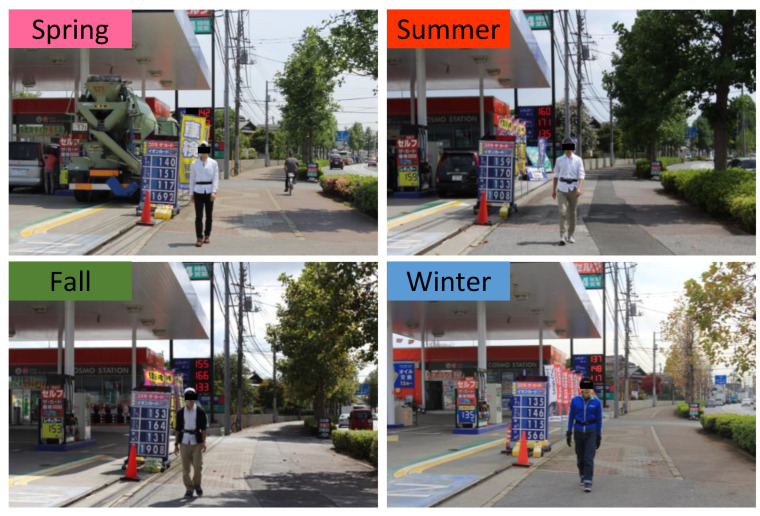
Experimental scene in the city area. The top left photo (spring), bottom left photo (fall), and bottom right photo (winter) are reprinted from Song et al. (2014) [20], Song et al. (2015) [21], and Song et al. (2013) [22], respectively.

The study was conducted in Kashiwa-no-ha Park in Kashiwa City, Chiba Prefecture, Japan. The site has a large pond at the center, and several hardwood trees are planted, such as maple, tulip, cherry, and chestnut. Thus, people can feel seasonal changes (Figure 1). The participants walked around its large pond. A city area around the urban park was selected as the control site. It was surrounded by housing complexes and commercial facilities (Figure 2). The experiments were commonly performed during sunny days. Table 1 shows the temperature, relative humidity, and intensity of illumination of the two experimental sites.

### 2.2. Participants

This research was conducted in accordance with the guidelines of the Declaration of Helsinki, and the protocol was approved by the Ethics Committees of the Center for Environment, Health and Field Sciences, Chiba University, Japan (project identification no. 5).

Twelve Japanese male university students participated in the summer experiment. They were recruited through snowball sampling. The inclusion criteria were healthy male university and graduate students aged 20–29 years, and the exclusion criterion was diagnosis of respiratory diseases (chronic rhinitis, asthma, etc.). For other diseases, a self-reporting system was adopted, and the experimenter conducted observation when explaining the ethics review before the experiment. The participants for experiments in other seasons were recruited using the same sampling method and inclusion and exclusion criteria. They were informed about the aims and procedures of the study prior to conducting the experiment. Moreover, informed consent was obtained. Consumption of alcohol, tobacco, and caffeine was prohibited during the study period.

Seasonal difference was analyzed using data obtained from 51 people (spring: n = 12, summer: n = 12, fall: n = 20, and winter: n = 7). The mean ± standard deviation of age, height, and weight were 22.2 ± 2.0 years, 172.1 ± 5.1 cm, and 63.4 ± 8.0 kg, respectively. In detail, the mean ± standard deviation of age, height, and weight were 21.1 ± 1.7 years, 173.5 ± 5.4 cm, and 62.7 ± 6.9 kg in spring; 22.3 ± 1.4 years, 170.4 ± 4.5 cm, and 64.9 ± 10.3 kg in summer; 22.3 ± 1.2 years, 171.1 ± 4.7 cm, and 62.3 ± 7.0 kg in fall; and 23.6 ± 4.0 years, 176.0 ± 5.1 cm, and 65.5 ± 8.7 kg in winter.

### 2.3. Experimental Procedure

Each subject walked in the urban park or city area for 15 min. A within-subject experimental design was used. Two participants were paired to eliminate the effect of the order of sites walked. One participant walked first in the urban park and then in the city area. Meanwhile, the other participant walked first in the city area and then in the urban park. After walking, they returned to the waiting room and completed several questionnaires. They rested for approximately 20 min and repeated the experiment in the opposite areas. The walking distance between the urban park and city area was the same, and the participants were instructed to walk slowly and at the same speed. There was no difference in the physiological indices before the start of each walk between the two environments. In addition, there were no significant differences with respect to the average walking speed of the participants between the two environments.

### 2.4. Physiological Measurements

Heart rate and heart-rate variability (HRV) were used as physiological indicators. HRV is effective in assessing the interplay between the sympathetic and parasympathetic nervous activities [26].

The periods between consecutive R waves (R–R intervals) assessed using a portable electrocardiogram (Activtracer AC-301A; GMS, Tokyo, Japan) were analyzed to obtain HRV and heart rate. The power levels of the low-frequency (LF, 0.04–0.15 Hz) and high-frequency (HF, 0.15–0.40 Hz) components of the HRV were calculated using the maximum entropy method with MemCalc/Win (GMS) [27]. To normalize the HRV parameters across participants, natural logarithmic-transformed values were used in the analysis [28]. The HF component of the HRV reflects the parasympathetic nervous activity and the LF/HF reflects the sympathetic nervous activity [29].

### 2.5. Data Analysis

All statistical analyses were performed using the Statistical Package for the Social Sciences software, version 20.0 (IBM Corporation, Armonk, NY, USA). In addition to the new summer experiments, data from three previous experiments were analyzed. The average heart rates, ln(HF) and ln(LF/HF), during the 15 min walking period in the two sites were used in the analysis. In each season, the values between walking in an urban park and a city area were compared using the paired *t*-test.

To show the effect of walking in an urban park, differences (between walking in an urban park and walking in a city area) were calculated. Then, differences according to season were compared using one-way analysis of variance and the post-hoc test (Tukey HSD test). A *p* value of <0.05 was considered statistically significant.

## 3. Results

In summer, heart rates were significantly lower while walking in an urban park than those while walking on a city street (92.3 ± 2.3 vs. 96.1 ± 2.7 bpm, *p* < 0.01). There were no statistically significant differences with respect to ln(HF) (3.5 ± 0.3 vs. 3.3 ± 0.3 lnms^2^, *p* > 0.05) and ln(LF/HF) (1.90 ± 0.14 vs. 1.89 ± 0.13, *p* > 0.05) between walking in an urban park and on a city street.

**Figure 3:**
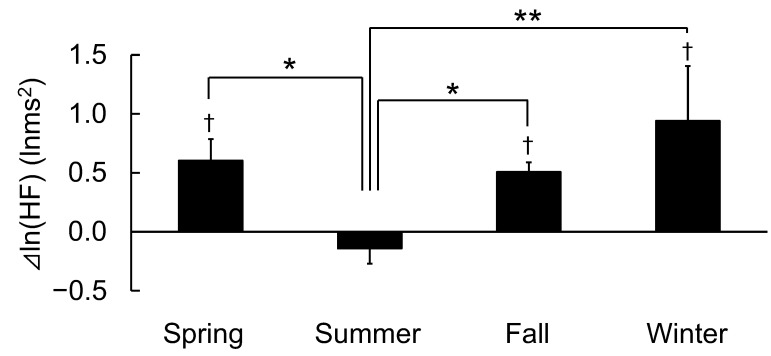
Differences in the changes in ln(HF) between walking in an urban park and a city area according to the four seasons. n = 51 (spring: n = 12, summer: n = 12, fall: n = 20, and winter: n = 7), mean ± standard deviation, †: *p* < 0.05 using the paired *t*-test (urban park vs. city area), *: *p* < 0.05, and **: *p* < 0.01 using one-way analysis of variance with the post-hoc test (four seasons).

Figure 3 shows the differences in the changes in ln(HF), which is an indicator of parasympathetic nervous system activity, between walking in an urban park and on a city street according to the four seasons.

The ln(HF) values during walking in an urban park were significantly higher compared to the city walking in spring, fall, and winter [20,21,22]. However, there was no significant difference in ln(HF) in summer (3.51 ± 0.29 vs. 3.65 ± 0.34 lnms^2^, *p* > 0.05). Based on these results, there were significant differences in seasonal changes (F(3,47) = 5.397, *p* < 0.01). Differences in ln(HF) by walking in an urban park were significantly higher in spring, fall, and winter compared with summer (spring: 0.60 ± 0.18 vs. summer: −0.14 ± 0.13, *p* < 0.05; fall: 0.51 ± 0.08 vs. summer, *p* < 0.05; and winter: 0.94 ± 0.46 vs. summer, *p* < 0.01).

**Figure 4:**
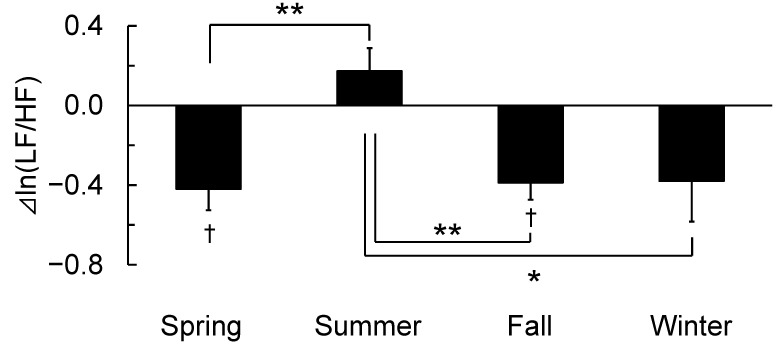
Differences in the changes in ln(LF/HF) walking in an urban park and in a city area according to the four seasons. n = 51 (spring: n = 12, summer: n = 12, fall: n = 20, and winter: n = 7); mean ± standard deviation, †: *p* < 0.05 using the paired *t*-test (urban park vs. city area); *: *p* < 0.05; and **: *p* < 0.01 using one-way analysis of variance with the post-hoc test (four seasons).

Figure 4 shows the differences in the changes in ln(LF/HF), which is an indicator of sympathetic nervous system activity, between walking in an urban park and in a city area according to the four seasons. The ln(LF/HF) during walking in an urban park was significantly lower compared to walking in the city area in spring and fall [20,21]. However, there was no significant difference with respect to ln(LF/HF) between participants who walked in an urban area and those who walked in a city area in summer (1.91 ± 0.15 vs. 1.74 ± 0.20, *p* > 0.05) and winter [22]. Therefore, a significant difference was observed in seasonal changes (F(3,47) = 5.979, *p* < 0.01). Differences in ln(LF/HF) by walking in an urban park were significantly lower in a city area in spring, fall, and winter compared with summer (spring: −0.42 ± 0.11 vs. summer: 0.17 ± 0.12, *p* < 0.01; fall: −0.39 ± 0.09 vs. summer, *p* < 0.01; and winter: −0.38 ± 0.20 vs. summer, *p* < 0.05).

**Figure 5:**
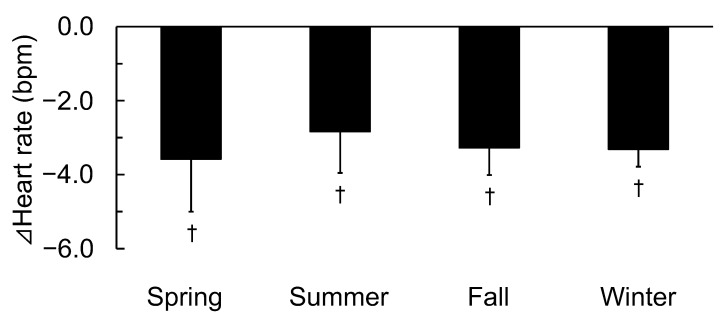
Differences in the changes in heart rate between walking in an urban park and walking in a city area according to the four seasons. n = 51 (spring: n = 12, summer: n = 12, fall: n = 20, and winter: n = 7); mean ± standard deviation, †: *p* < 0.05 using the paired *t*-test (urban park vs. city area).

Figure 5 shows the differences in the changes in heart rate between walking in an urban park and in a city area according to the four seasons. The heart rate during walking in an urban park was significantly lower compared to walking in the city area in all seasons [20,21,22] including summer (92.1 ± 3.2 vs. 95.0 ± 3.5 bpm, *p* < 0.05). However, there were no significant differences in seasonal changes (F(3,47) = 0.082, *p* > 0.05).

## 4. Discussion

In summer, the heart rate decreased significantly. However, there was no significant change in the parasympathetic and sympathetic nervous system activities. This was not in accordance with the results in other seasons [20,21]. The thermal environment is thought to have a highly influential effect. In winter, the body’s stress response caused by the cold can be reduced by wearing clothes, but in summer, the outdoor heat cannot be controlled. Thus, the relaxation effect may have been lower in summer than in other seasons.

The physiological responses to walking in urban parks according to the four seasons are as follows: the parasympathetic nervous system activity of the participants increased in all seasons except summer. Moreover, the participants had decreased sympathetic nervous system activity in spring and fall and decreased heart rate in all seasons.

Our results are almost consistent with those of previous studies that assessed the physiological effects of spending time in the forest environment [30,31]. These studies revealed that a simple walk or a rest in the forest can promote physiological relaxation as represented by increased parasympathetic nervous system activity and decreased sympathetic nervous system activity and heart rate. The current study had a similar result; that is, the effects of walking in urban green areas are similar to those of walking in forest areas.

In addition, walking in spring, fall, and winter had a greater physiological relaxation effect than walking in summer. Walking in spring and fall had the highest physiological relaxation effect. If people can well maintain warmth even in winter, walking can have physiological benefits.

Only few studies have assessed the effects of walking according to seasonal differences in urban green spaces. Cohen et al. [32] showed the climatic conditions between summer and winter in various urban parks with different vegetation coverage and their impacts on human thermal sensation. The results showed that an urban park with a dense canopy of trees has a maximum cooling effect during summer. However, it may have a negative effect on human thermal comfort in winter. By contrast, because it is hot during summer and cold during winter in lawn parks, the establishment of treed open spaces is important as they are most effective in mitigating heat. Yang et al. [33] showed the impacts of the composition and configuration of urban green spaces on urban thermal environment in all four seasons. However, these studies did not examine their direct effect on humans.

This research included male participants aged 20–29 years with a limited sample size and provided novel evidence on seasonal differences in physiological responses to walking in urban parks. The results provide helpful information on the use of parks in urban planning.

A recent study reported that middle-aged and older adults walking in an urban park exhibited lower heart rates and blood pressure [34]. These results suggest that walking in an urban park may be beneficial not only for young men but also for older people. To generalize our findings, studies with female participants, different age groups, and larger sample sizes should be conducted.

There is evidence supporting the beneficial health effects (e.g., improved HRV and decreased anxiety) of repeated moderate-intensity walking session in these environments [35]. In future studies, the effects of long-term and repetitive walking in urban parks should be reviewed in various ways, and attention should also be paid to the advantages of various activities in green spaces.

In addition, factors affecting seasonal changes, which have caused differences in physiological responses, must be validated.

## 5. Conclusions

The present study showed seasonal differences in physiological responses to walking in urban parks; that is, the participants had increased parasympathetic nervous system activity in all seasons except summer. Moreover, they had decreased sympathetic nervous system activity in spring and fall, during which people experienced a positive feeling while walking. The heart rate of the participants decreased in all seasons. We conclude that walking in spring, fall, and winter had a greater physiological relaxation effect than walking in summer.

## Figures and Tables

**Table 1:** Details of the experimental site.

	Spring		Summer		Fall		Winter	
Experimental day	15 May 2013 16 May 2013 17 May 2013		30 July 2014 31 July 2014		07 October 2014 15 October 2014 16 October 2014		21 November 2012 23 November 2012 24 November 2012	
Experimental site	UP	CA	UP	CA	UP	CA	UP	CA
Temperature (°C)	27.0	24.7	31.3	35.6	18.0	19.2	13.8	14.0
Relative humidity (%)	37.3	39.2	53.8	40.3	71.5	64.7	50.9	52.1
Illumination (lx)	80,730	4990	3550	62,890	24,230	38,870	7930	8430

UP, urban park; CA, city area.

## Data Availability

The data that support the finding of this study are available from the corresponding author upon reasonable request.
